# Supplementary material for: Transposase-assisted target-site integration for efficient plant genome engineering
Source: Nature. 2024 Jun 26;631(8021):593–600. doi: 10.1038/s41586-024-07613-8 (PMC11254759; doi:10.1038/s41586-024-07613-8)
Supplement: Supplementary file 1 — Full scanned images of western blots from Extended Data Fig. 4b [file 41586_2024_7613_MOESM1_ESM.pdf]

nature portfolio
https://doi.org/10.1038/s41586-024-07613-8

# Supplementary information

# Transposase-assisted target-site integration for efficient plant genome engineering

In the format provided by the
authors and unedited

# Supplemental Fig. 1

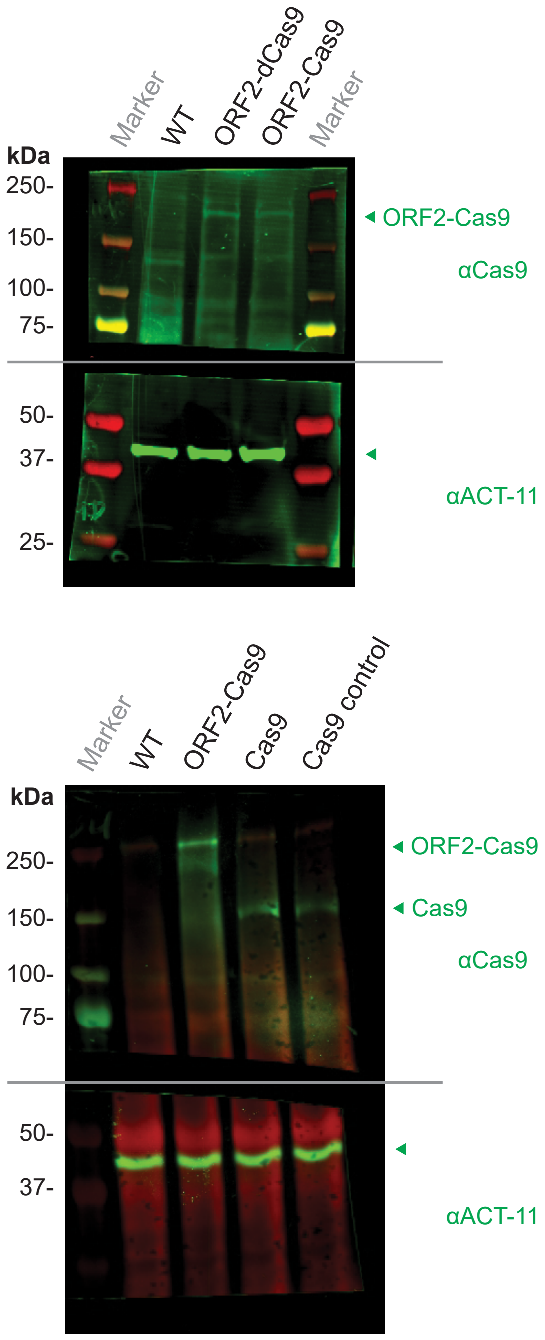


Used in Extended Data Fig. **4b**
