# Supplementary material for: Transposase-assisted target-site integration for efficient plant genome engineering
Source: Nature. 2024 Jun 26;631(8021):593–600. doi: 10.1038/s41586-024-07613-8 (PMC11254759; doi:10.1038/s41586-024-07613-8)
Supplement: Supplementary file 2 — Reporting Summary [file 41586_2024_7613_MOESM2_ESM.pdf]

nature portfolio

Corresponding author(s): R. Keith Slotkin

Last updated by author(s): Mar 11, 2024

Reporting Summary

Nature Portfolio wishes to improve the reproducibility of the work that we publish. This form provides structure for consistency and transparency in reporting. For further information on Nature Portfolio policies, see our Editorial Policies and the Editorial Policy Checklist.

Statistics

For all statistical analyses, confirm that the following items are present in the figure legend, table legend, main text, or Methods section.

n/a	Confirmed	
☐	☒	The exact sample size (*n*) for each experimental group/condition, given as a discrete number and unit of measurement
☐	☒	A statement on whether measurements were taken from distinct samples or whether the same sample was measured repeatedly
☒	☐	The statistical test(s) used AND whether they are one- or two-sided<br>*Only common tests should be described solely by name; describe more complex techniques in the Methods section.*
☒	☐	A description of all covariates tested
☐	☒	A description of any assumptions or corrections, such as tests of normality and adjustment for multiple comparisons
☐	☒	A full description of the statistical parameters including central tendency (e.g. means) or other basic estimates (e.g. regression coefficient) AND variation (e.g. standard deviation) or associated estimates of uncertainty (e.g. confidence intervals)
☒	☐	For null hypothesis testing, the test statistic (e.g. *F*, *t*, *r*) with confidence intervals, effect sizes, degrees of freedom and *P* value noted<br>*Give P values as exact values whenever suitable.*
☒	☐	For Bayesian analysis, information on the choice of priors and Markov chain Monte Carlo settings
☒	☐	For hierarchical and complex designs, identification of the appropriate level for tests and full reporting of outcomes
☒	☐	Estimates of effect sizes (e.g. Cohen's *d*, Pearson's *r*), indicating how they were calculated

Our web collection on statistics for biologists contains articles on many of the points above.

Software and code

Policy information about availability of computer code

Data collection	Illumina BaseSpace software v7.30
Data analysis	High Resolution Melt Software v2.0<br>cutadapt v4.1<br>bwa v0.7.17-r1194-dirty<br>gatk v3.5-0-g36282e4<br>ggplot2 v3.3.6<br>bbmap v39.01 (this covers the bbduk clumpify.sh and dedupe.sh)<br>bowtie2 v2.4.4<br>CMplot v4.2.0<br>Benchling Biology Software v1<br>The perimeters, variables and sequences used for each program are described in the Methods section.

For manuscripts utilizing custom algorithms or software that are central to the research but not yet described in published literature, software must be made available to editors and reviewers. We strongly encourage code deposition in a community repository (e.g. GitHub). See the Nature Portfolio guidelines for submitting code & software for further information.

Data

Policy information about availability of data

All manuscripts must include a data availability statement. This statement should provide the following information, where applicable:

- Accession codes, unique identifiers, or web links for publicly available datasets
- A description of any restrictions on data availability
- For clinical datasets or third party data, please ensure that the statement adheres to our policy

There are no restrictions on the presented data. Amplicon-sequencing and Insertion-seq data from Fig. 2 and 4 for Arabidopsis and soybean are provided via the NCBI sequence read archive as GSE227105. Genome sequences and annotations used come from TAIR10 (Columbia ecotype Arabidopsis)(https://www.arabidopsis.org/download/) and Williams 82 Wm82.a4.v1 from Phytozome (soybean)(https://phytozome-next.jgi.doe.gov/info/Gmax_Wm82_a4_v1).

Human research participants

Policy information about studies involving human research participants and Sex and Gender in Research.

Reporting on sex and gender	N/A
Population characteristics	N/A
Recruitment	N/A
Ethics oversight	N/A

Note that full information on the approval of the study protocol must also be provided in the manuscript.

Field-specific reporting

Please select the one below that is the best fit for your research. If you are not sure, read the appropriate sections before making your selection.

☒ Life sciences ☐ Behavioural & social sciences ☐ Ecological, evolutionary & environmental sciences

For a reference copy of the document with all sections, see nature.com/documents/nr-reporting-summary-flat.pdf

Life sciences study design

All studies must disclose on these points even when the disclosure is negative.

Sample size	For pools of seedlings analyzed, pools are between 30 and 50 individuals. For the targeted insertion experiment, the number of plants tested is indicated on Figure 3C and 4B. The n= is indicated above the data. No size calculation was performed but rather the sample size was dictated by the number of transgenic plants generated by the experiment. These numbers are sufficient to minimize the effects of ranomd transgene insertion position.
Data exclusions	None
Replication	Biological replicates (non-overlapping) were used for the deep sequencing data in Fig. 2E-F and Extended Data Fig. 6A-B.
Randomization	Samples were allocated into groups based on their genotype and transgene. Within these genotype groups, samples were given random available sequencing indexes. Otherwise, randomization was not relevant to our study.
Blinding	Blinding was not relevant to this study because no clinical research was performed.

Reporting for specific materials, systems and methods

We require information from authors about some types of materials, experimental systems and methods used in many studies. Here, indicate whether each material, system or method listed is relevant to your study. If you are not sure if a list item applies to your research, read the appropriate section before selecting a response.

Materials & experimental systems

n/a	Involved in the study	
☐	☒	Antibodies
☐	☒	Eukaryotic cell lines
☒	☐	Palaeontology and archaeology
☒	☐	Animals and other organisms
☒	☐	Clinical data
☒	☐	Dual use research of concern

Methods

n/a	Involved in the study	
☒	☐	ChIP-seq
☒	☐	Flow cytometry
☒	☐	MRI-based neuroimaging

Antibodies

Antibodies used

Primary antibodies: anti-Actin (Agrisera, AS10 702) and anti-Cas9 (Diagenode, C15310258-100). Secondary antibodies: anti-Act 11: AzureSpectra, goat anti-mouse 800, AC2135; and anti-Cas9: AzureSpectra goat anti-rabbit 800, AC2134

Validation

Anti-Actin (Agrisera, AS10 702) is standard in the field as a loading control for Arabidopsis protein on Western blots and is validated at https://www.citeab.com/antibodies/662899-as10-702-anti-actin-11. Anti-Cas9 (Diagenode, C15310258-100) is validated for Cas9 in Arabidopsis in Extended Data Fig. 4b. Cas9 signal is not detected in the WT Arabidopsis plants that do not have a Cas9 transgene.

Eukaryotic cell lines

Policy information about cell lines and Sex and Gender in Research

Cell line source(s)

BY4741 yeast strain

Authentication

Describe the authentication procedures for each cell line used OR declare that none of the cell lines used were authenticated.

Mycoplasma contamination

Confirm that all cell lines tested negative for mycoplasma contamination OR describe the results of the testing for mycoplasma contamination OR declare that the cell lines were not tested for mycoplasma contamination.

Commonly misidentified lines
(See ICLAC register)

Name any commonly misidentified cell lines used in the study and provide a rationale for their use.
